# Supplementary material for: Autonomous learning of features for control: Experiments with embodied and situated agents
Source: PLoS One. 2021 Apr 15;16(4):e0250040. doi: 10.1371/journal.pone.0250040 (PMC8049238; doi:10.1371/journal.pone.0250040)
Supplement: Attachment — Submitted filename: reply-to-reviewers.docx [file pone.0250040.s001.docx]

Please include the following items when submitting your revised manuscript:

- A rebuttal letter that responds to each point raised by the academic editor and reviewer(s). You should upload this letter as a separate file labeled 'Response to Reviewers'.
- A marked-up copy of your manuscript that highlights changes made to the original version. You should upload this as a separate file labeled 'Revised Manuscript with Track Changes'.
- An unmarked version of your revised paper without tracked changes. You should upload this as a separate file labeled 'Manuscript'.

**Reviewer #1:**

**This paper describes a a method of simultaneously training a feature extracting network alongside a control network. They validate their results across several well known benchmark problems. Although I am not a domain expert the paper was quite well written and relatively easy to understand.**

We are glad you appreciated the paper and the presentation.

**Reviewer #2:**

Overall comments:

**1. 'Episodes last 1,000 steps in the case of the Walker2DBullet, HalfCheetahBullet, and BipedalWalkerHardcore problems, and 10,000 steps in the case of the MIT racecar'. It is not clear how this is derived, is it based on past research or experimentally determined? Detailed justification of why MIT needs more steps is expected.**

1000 steps is commonly used for the first four problems since it sufficient to evaluate the capability of the agents to walk and, in the case of the BipedalWalkerHardcore, to overcome obstacles. In the case of the MIT racecar we used longer evaluation episodes since agents require at least 7000 steps to complete one lap of the environment. We explicated this in the revised manuscript

**2. 'Data computed by post-evaluating the best individual of each generation for 3 episodes.' , is a confusing statement that needs to be made clear, preferable with including a table to explain results on figure 3. Each episode terminated after 1000 steps, however it is not clear how 1e7 steps equate to the 3 episodes.**

Evolving agents are evaluated for 1 episode lasting 1000 steps in the case of the Walker2DBullet, HalfCheetahBullet, for 5 episode lasting 1000 steps for a total of 5000 steps in the case of the BipedalWalkerHardcore, and for 1 episode lasting 10,000 steps in the case of the MIT race-car.

The evolutionary process is continued until the total number of steps performed exceeds 5 * 10^7^ (20 * 10^7^ in the case of the BipedalWalkerHardcore which requires more time than the other problems to achieve good performance.

The best agent of each generation are post-evaluated for 3 episodes (20 episodes in the case of the BipedalWalkerHardcore). The purpose of post-evaluation is that to estimate more accurately the performance of the evolving agents. The duration of the post-evaluation is not related to the duration of the evolutionary process. This is the reason why they differ. The duration of the evolutionary process is the total number of steps performed by evolving individuals of different generations. The duration of the post-evaluation is the number of steps performed by a single individual during its post-evaluation.

We explained the reason why we used a greater number of evaluation episodes and whether we continued the evolutionary process for a longer period of time in the case of certain problems. Moreover, we explained the purpose and the modality of the post-evaluation in the revised version of the manuscript.

**3. The variable 's' should be defined in Figure 2 as done with the rest of other variables for clarity.**

done

**4. Discussion of the results is too brief, expected some postulations and thorough discussion on why the sequence-to-sequence model outperforms the other feature extracting methods used in previous works.**

done

**5. Limitations of this research and future direction of the study is not presented in this article.**

done

Thank you for your constructive comments. **Several typos to be attended as given below:**

We fixed them
